# C-terminal amino acids are essential for human heat shock protein 70 dimerization

**DOI:** 10.1007/s12192-014-0526-3

**Published:** 2014-07-17

**Authors:** Guillaume Marcion, Renaud Seigneuric, Evelyne Chavanne, Yves Artur, Loïc Briand, Tarik Hadi, Jessica Gobbo, Carmen Garrido, Fabrice Neiers

**Affiliations:** 1 INSERM, UMR 866, 7 blvd Jeanne d’Arc, 21000 Dijon, France; 2 Université de Bourgogne, Esplanade Erasme, Dijon, France; 3 Centre des Sciences du Goût et de l’Alimentation, INRA UMR 1324, CNRS UMR 6265, Université de Bourgogne, Dijon, France; 4 Anticancer Center Georges François Leclerc, Dijon, France; 5 CSGA, 17 rue Sully, 21000 Dijon, France

**Keywords:** Hsp70, HSPA1A, Dimer, Monomer, Cancer

## Abstract

The human inducible heat shock protein 70 (hHsp70), which is involved in several major pathologies, including neurodegenerative disorders and cancer, is a key molecular chaperone and contributes to the proper protein folding and maintenance of a large number of protein structures. Despite its role in disease, the current structural knowledge of hHsp70 is almost exclusively based on its *Escherichia coli* homolog, DnaK, even though these two proteins only share ~50 % amino acid identity. For the first time, we describe a complete heterologous production and purification strategy that allowed us to obtain a large amount of soluble, full-length, and non-tagged hHsp70. The protein displayed both an ATPase and a refolding activity when combined to the human Hsp40. Multi-angle light scattering and bio-layer interferometry analyses demonstrated the ability of hHsp70 to homodimerize. The role of the C-terminal part of hHsp70 was identified and confirmed by a study of a truncated version of hHsp70 that could neither dimerize nor present refolding activity.

**Electronic supplementary material:**

The online version of this article (doi:10.1007/s12192-014-0526-3) contains supplementary material, which is available to authorized users.

## Introduction

Molecular chaperones assist in the non-covalent folding or unfolding of a polypeptide chain, refolding of misfolded intermediates, the assembly or disassembly of a complex, and its proper localization in the cell (Ellis 1987; Fink 1999; Taipale et al. 2010). Many cellular proteins tend to aggregate and become insoluble after a stress such as a heat shock, and the molecular chaperones that play a role in this process include members of the heat shock protein (Hsp) family. Hsps are structurally and functionally diverse proteins and are classified according to their approximate molecular mass. These proteins include six families: small Hsps, Hsp40, Hsp60, Hsp70, Hsp90, and Hsp110 (Brodsky and Chiosis 2006; Garrido et al. 2006; Mayer and Bukau 2005; Powers and Workman 2007; Schmitt et al. 2006). The human Hsp70 family is encoded by 13 genes members (excluding the pseudogenes) (Brocchieri et al. 2008). At the protein level, the constitutively-expressed Hsc70 and the inducible Hsp70 share nearly 80 % amino acid sequence identity and differ by their C-terminal region (Dworniczak and Mirault 1987; Jinwal et al. 2013; Maeda et al. 2007). Present at low or undetectable levels in most unstressed normal cells and tissues, Hsp70 (also referred to as Hsp72, Hsp70-1, or HspA1A) is among the most stress-inducible molecular chaperones (Dworniczak and Mirault 1987; Freeman et al. 1995). This ~70 kDa chaperone interacts with Hsp40 to accommodate its substrates, binds to short linear stretches of hydrophobic residues and refolds its substrate properly through cycles of ATP binding, hydrolysis, and release (Ellis et al. 2007; Schlecht et al. 2011; Taipale et al. 2010). Acting within the chaperone network, Hsp70 is central in protein folding and homeostasis (Bukau et al. 2000; Hartl et al. 2011; Hartl and Hayer-Hartl 2002). Human Hsp70 (hHsp70) is involved in several pathologies, including neurodegenerative disorders and cancer, where it exerts both a chaperone and an anti-apoptotic function (Garrido et al. 2001; Hartl et al. 2011; Jaattela 1999; Seigneuric et al. 2011b). The modulation of hHsp70 activity may thus represent an interesting therapeutic approach for some of these pathologies (Calderwood 2010, 2013; Gobbo et al. 2011; Jego et al. 2010; Seigneuric et al. 2011a; Stangl et al. 2011). Despite the importance and relevance of hHsp70 in health and disease, the most mechanistic information has been inferred from comparisons with its bacterial homolog, DnaK (Bhattacharya et al. 2009; Kityk et al. 2012; Kumar et al. 2011; Swain et al. 2007; Taipale et al. 2010; Vogel et al. 2006), despite the fact that human and bacterial Hsp70 share only ~50 % amino acid sequence identity (Hunt and Morimoto 1985; Nicolaï et al. 2013; Zhu et al. 1996). The hHsp70 is divided into two functional domains connected by a short linker: an amino-terminal nucleotide binding domain (NBD) and a carboxyl-terminal substrate binding domain (SBD). Allosteric regulation of hHsp70 between these two domains remains to be elucidated (Nicolaï et al. 2013). In addition, there are no full-length structures of the members of the hHsp70 protein family available, most likely due to the limited amount of pure available protein and/or technical challenges. Therefore, current structural representations of hHsp70 are based on the full-length structure of *Escherichia coli* DnaK solved by NMR (PDB ID: 2KHO) (Bertelsen et al. 2009). More recently, full-length *E. coli* DnaK structures in an ATP-bound state were solved using X-ray diffraction (PDB ID: 4B9Q, and 4JNE) (Kityk et al. 2012; Qi et al. 2013). Thus, most of the information on hHsp70 is derived from the *E. coli* Hsp70 homolog DnaK. A few independent studies have revealed a mixture of monomeric and dimeric forms after purification, but the in vivo relevance of these forms is a debatable point (Palleros et al. 1993; Richarme and Kohiyama 1993). Using electron microscopy, Thompson et al. demonstrated that a DnaK mixture of dimer and monomer led to monomerization following substrate addition (Thompson et al. 2012). With regards to hHsp70, different groups reported the existence of oligomeric states from monomer to high order oligomers (Angelidis et al. 1999; Aprile et al. 2013; Nemoto et al. 2006). A variety of mechanisms have been proposed to explain the change of oligomeric state, including cystein oxidation-dependence (Nemoto et al. 2006) or temperature dependency (Angelidis et al. 1999). Recently, an interaction between the linker and the SBD has been proposed to mediate the oligomerization of hHsp70 (Aprile et al. 2013). Protein quantity is still a limiting step for biophysical or structural studies allowing for the characterization of the oligomeric state. To date, only commercial hHsp70 (HspA1A), sold by Stressgen, is available; therefore, the process of purification remains confidential. The heterologous expression and purification of the chaperone family proteins tends to exhibit low recovery and specificity (Nicoll et al. 2006). Some studies report a partial purification of hHsp70 fused to another protein (Macejak et al. 1990) or to a His-tagged protein (Aprile et al. 2013; Nemoto et al. 2006). Moreover, it has been demonstrated that small N- or C-termini extensions of the rat Hsc70 homolog or hHsp70 may alter their ATPase activities and peptide binding (Boice and Hightower 1997). In the present paper, a detailed methodology allowing for the production of a large quantity of a recombinant, pure, full-length, and fully active hHsp70 is described. This approach enabled us to explore, for the first time, the oligomeric state of a non-tagged hHsp70 using an improved size exclusion chromatography (SEC) technology that can provide an absolute molecular mass: SEC-MALS (multi-angle light scattering). Using additional biophysical, and biochemical studies, we demonstrate the molecular basis driving the formation of the observed dimeric state.

## Results

### Characterization of the full-length hHsp70 and the delta-hHsp70

**Fig. 1:**
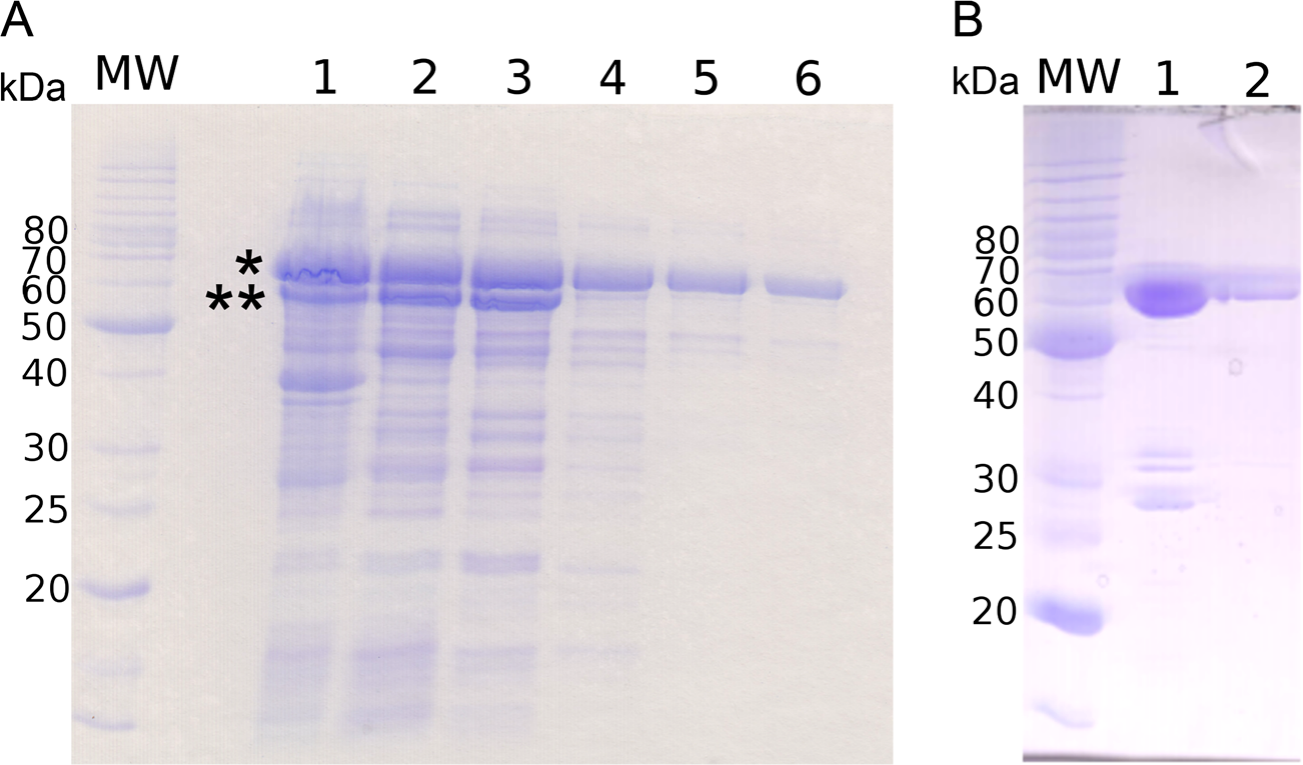
Purification of full-length hHsp70 and delta-hHsp70. **a** Proteins were separated by 12 % SDS-polyacrylamide gel electrophoresis and stained with Coomassie blue. The *different lanes* correspond to cell pellets 12 h after IPTG induction (*lane 1*), supernatant after sonication and centrifugation (*lane 2*), pool after the DEAE exchange column chromatography (*lane 3*), pool after the Q-sepharose exchange column chromatography (*lane 4*), pool after the Superdex 200 chromatography (*lane 5*), and after the final Q-sepharose exchange column chromatography (*lane 6*). The *star and double star* indicate full-length hHsp70 and delta-hHsp70, respectively. **b** The pool of delta-hHsp70 after the DEAE exchange column chromatography (*lane 1*) and after purification on exclusion chromatography using a Superdex 200 (*lane 2*) was analyzed by SDS-PAGE. *MW* molecular mass markers

A synthetic, codon-optimized coding sequence for hHsp70 expression was introduced in a pET21a plasmid. Among the different cells that were tested for pET21-hHsp70 plasmid expression, BL21 Star (DE3) presented the highest level of expression. After transformation and production, the samples were analyzed by sodium dodecyl sulfate polyacrylamide gel electrophoresis (SDS-PAGE). Two bands were observed after induction: an intense band that migrated at approximately 70 kDa (full-length hHsp70) and a second, less intense band that migrated at approximately 60 kDa (delta-hHsp70) (Fig. 1a, lane 1).

Peptide mass fingerprinting, involving tryptic cleavage combined with MALDI-ToF analysis, confirmed that the 70 kDa protein corresponded to the full-length hHsp70 (Supplemental figure 1 A and C). All peptide fragments corresponded to hHsp70. This analysis also showed that the contamination from endogenous DnaK was non-detectable or absent. Moreover, these fragments covered the entire protein length.

During production, hHsp70 showed a sensitivity to a specific proteolysis, resulting in a band localized at approximately 60 kDa (delta-hHsp70) (Fig. 1, panel a). This band was observed in all of the different production and purification batches tested, similar to other studies where Hsp70 was produced (Gross and Hessefort 1996; Peake et al. 1998). Mass spectroscopy fingerprinting analysis of this band showed a panel of peptides originating from hHsp70, thus proving that the pure protein obtained here corresponds to a truncated form of the hHsp70. The peptide analysis (Supplemental figure 1 B and D) revealed the presence of peptide fragments from the third amino acid residue. Moreover, none of the fragments were observed after the K561 amino acid residue, thereby suggesting a protein mass of 61.4 kDa and a proteolysis cleavage occurring after the K561 amino acid residue.

### Purification of full-length hHsp70 and delta-hHsp70

Protein purity after the different purification steps is shown in Fig. 1, panel a. After the centrifugation of sonicated cells, two thirds of the total recombinant hHsp70 was found in the pellet and one third was found in the supernatant. Different refolding strategies were carried out on the inclusion bodies without success because the resulting soluble protein showed no functional activity in the tested conditions (data not shown). The soluble hHsp70 obtained after the first centrifugation (supernatant) was loaded on an anion exchange resin column (DEAE resin). Interestingly, this step allowed us to separate the two hHsp70 forms observed after production. Delta-hHsp70 eluted at approximately 0.15 mM KCl, whereas the full-length hHsp70 eluted at 0.20 M KCl. The latter value is in agreement with the low theoretical isoelectric pH of 5.5 calculated for the full-length hHsp70. This observation also suggests a higher value of the isoelectric pH for delta-hHsp70. A simulated truncation of hHsp70 leading to a 60 kDa protein showed that only a C-terminal truncation of the protein increases the isoelectric pH by 0.3 pH units, whereas an N-terminal truncation decreases this value by 0.2 pH units. These isoelectric pH differences between the two forms are also in agreement with the mass spectroscopy experiment, suggesting a truncation occurring after the K561 residue.

Fractions containing hHsp70 were pooled and loaded onto a strong anion exchange resin (Q-sepharose resin) followed by size exclusion chromatography (Superdex 200 Hiload 26/60). Next, the pooled fractions were reloaded on the strong anion exchange resin (Q-sepharose resin). Samples of each purification step were loaded on a 12.5 % SDS-PAGE (Fig. 1, panel a). Thirty milligrams of pure hHsp70 was obtained per liter of culture, corresponding to a purification efficiency of approximately 15 % of the total hHsp70 produced (soluble and non-soluble).

With regards to delta-hHsp70 separated after the first anion exchange resin column, this protein sample was concentrated to a 5-mL final volume and loaded on a gel filtration column (Superdex 200 Hiload 26/60). Again, sample of each purification steps were loaded on a 12.5 % SDS-PAGE (Fig. 1, panel b).

After 12 months, the full-length hHsp70 was again tested without signs of protein precipitation. Further, its folding activity was similar to the freshly produced protein, thereby suggesting that long-term storage did not alter the chaperone activity.

### Intrinsic tryptophan fluorescence emission

**Fig. 2:**
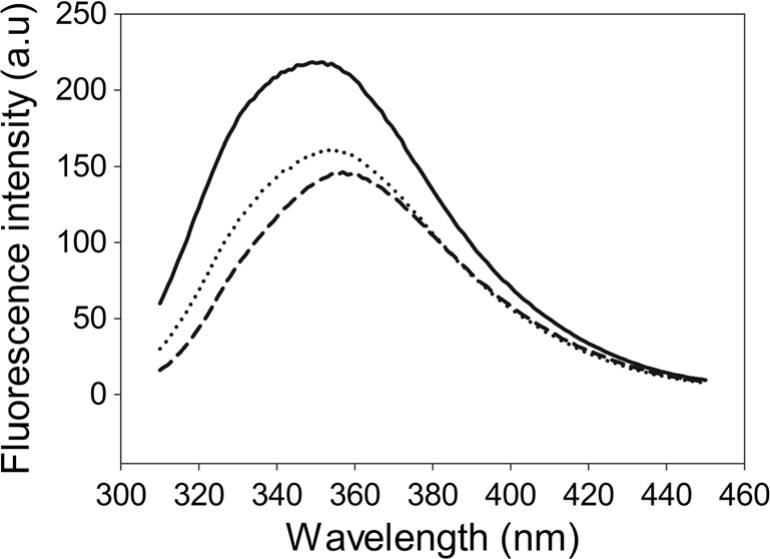
Fluorescence emission spectra of full-length hHsp70 with or without denaturation. The spectra of 5 μM protein were measured in 20 mM Tris–HCl pH 8.0 buffer (*solid curve*), in the same buffer supplemented with 2 M of guanidine chloride (*dotted curve*), or in 4 M of guanidine chloride (*dashed curve*). After excitation at 295 nm, the measured maximum emission spectra were 349, 354, and 356 nm (*red shift*), respectively, while the maximum of fluorescence emission spectra decreased. One representative curve is shown (*n* = 4)

To check the quality of the full-length hHsp70, we recorded its tryptophan (Trp) fluorescence emission spectrum before and after denaturation with two concentrations of guanidine chloride. To select a typical Trp fluorescence emission, a 295-nm excitation wavelength was used. Full-length hHsp70 contains two Trp (amino acid position 90 and 580). The maximum emission fluorescence signal due to the Trp90 and Trp580 amino acid residues at 349 nm supports their polar environment location (Fig. 2). After being exposed to a 2- and 4-M guanidine chloride solution, the full-length hHsp70 maximum emission spectra (at 354 and 356 nm, respectively) presented a red shift compared to the non-denatured protein, typical of an increased solvent exposure (Fig. 2). The red shift and the observed maximum emission intensity decrease demonstrated a full-length hHsp70 denaturation. The observations support that the protein was initially properly folded in the starting solution before denaturation.

### Secondary structure analysis of recombinant full-length hHsp70 and delta-hHsp70

**Fig. 3:**
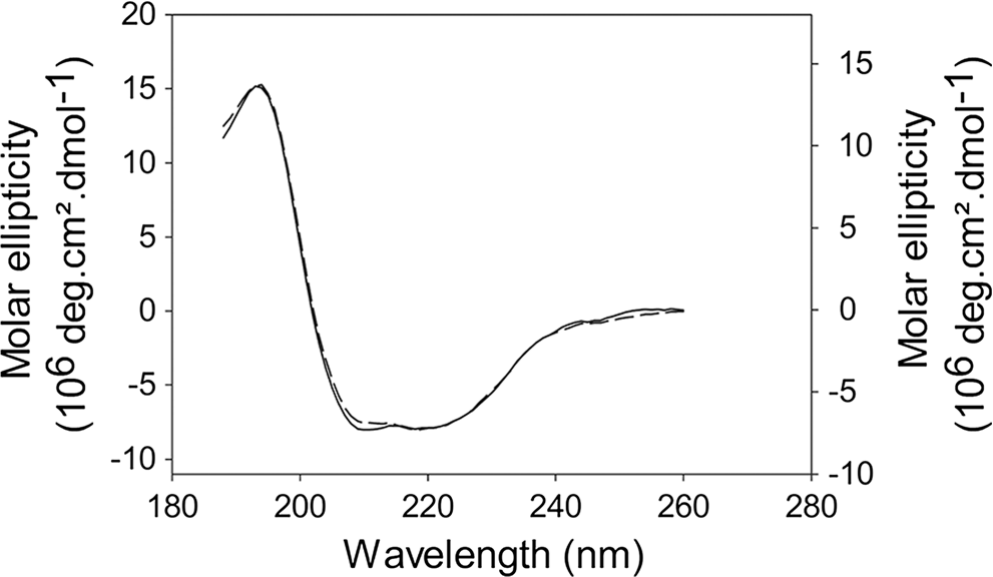
Secondary structure analysis by circular dichroism. Spectra of the full-length hHsp70 (*solid curve*, *left axis*) and delta-hHsp70 (*dashed curve*, *right axis*) are presented in molar ellipticity (deg.cm^2^.dmol^−1^). The spectra were recorded at 20 °C in a 0.01-cm path cell length with a 5-μM protein concentration in a 20-mM Tris–HCl pH 8.0 buffer

To confirm the folding of full-length hHsp70 and delta-hHsp70, we carried out circular dichroïsm spectrophotometer analysis. The recorded signals between 188 and 260 nm were superposed (Fig. 3). The good correlation of the two spectra supports a similar folding of the full-length and truncated hHsp70 forms. The far-UV spectra of the two proteins displayed a positive peak centered at 193 nm and two negative peaks at 210 and 218 nm, showing the helical character of both proteins. The deconvolution of the circular dichroïsm (CD) spectra revealed that the full-length hHsp70 was composed of approximately 50–60 % alpha-helix and 25–30 % beta-sheet, whereas the truncated form revealed 40–50 % alpha-helix and 50 % beta-sheet.

### Oligomeric state analysis of recombinant full-length hHsp70 and delta-hHsp70

**Fig. 4:**
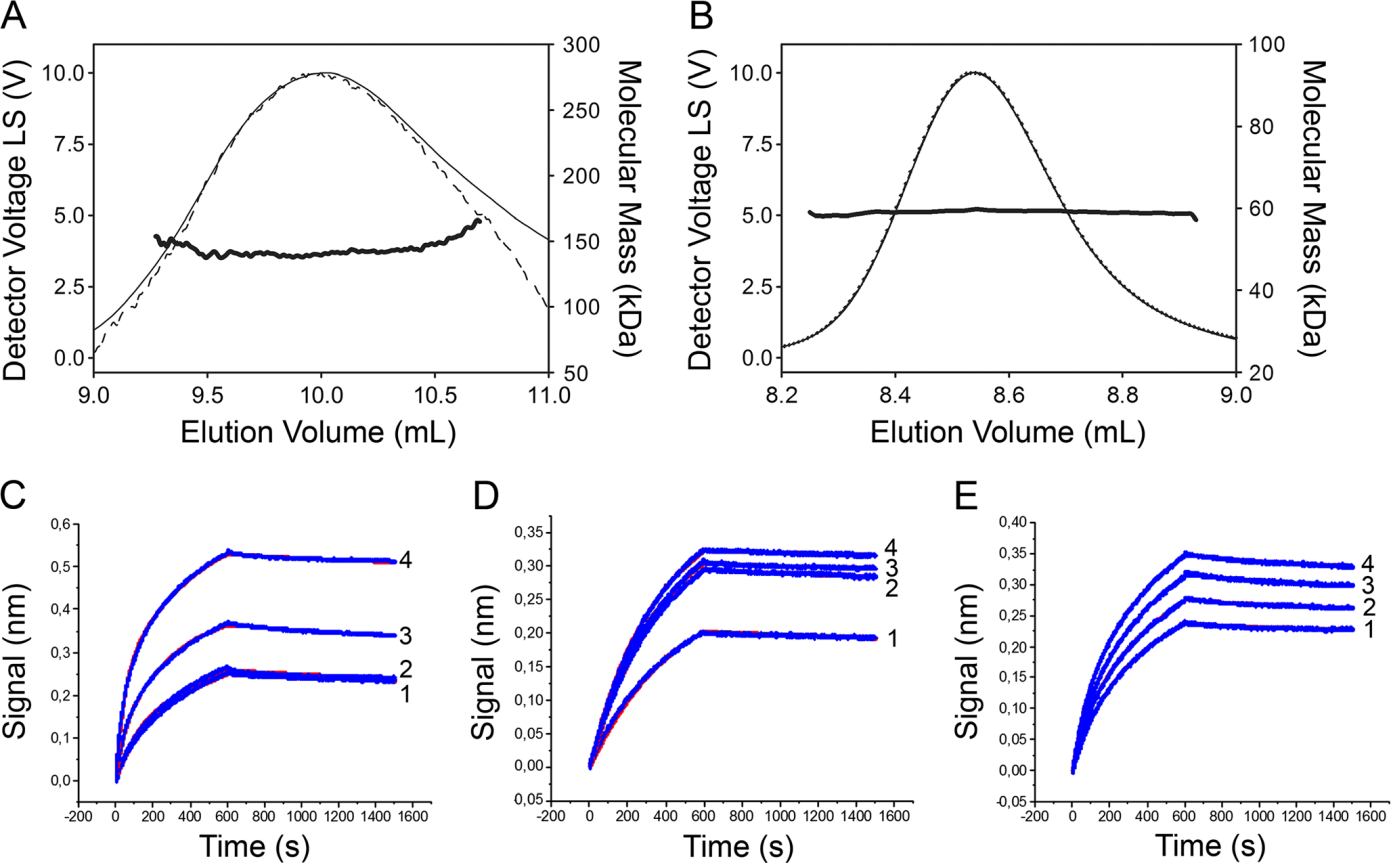
Oligomeric state analysis of hHsp70. After size exclusion chromatography (*SEC*) and separation with a suitable chromatography column (MP015S5 column from Wyatt for delta-hHsp70, and MP030S5 column from Wyatt for the full-length hHsp70), the molecular mass was determined from the Raleigh ratio, measured by static light scattering and the refractive index. The calculated molecular mass (*bold black curve*), refraction index (*dotted black curve*), and light scattering (*black curve*) are shown. The computed molecular masses are as follows: 139 kDa (full-length hHsp70: dimer) (**a**), and 59 kDa (delta-hHsp70: monomer) (**b**). **c**, **d,** and **e** Sensorgrams were obtained by Bio-Layer Interferometry (*BLI*). The different concentrations of the analytes in PBS are: panel **c** hHsp70 (1: 200, 2: 300, 3: 400, and 4: 500 nM); panel **d** hHsp40 (1: 20, 2: 30, 3: 40, and 4: 50 nM); and panel **e** DnaK (1: 200, 2: 300, 3: 400 and 4: 500 nM). The best fit is provided with the 1:1 model (*red fit*) with a K_D_ value of 4.5 +/− 0.1 nM, a r^2^ of 0.99 for hHsp70:hHsp70 (**c**), a K_D_ value of 0.52 +/− 0.01 nM, a r^2^ of 0.99 for hHsp70:hHsp40 (**d**), and a K_D_ value of 5.4 +/− 0.2 nM, a r^2^ of 0.99 for DnaK:DnaK (**e**)

Using size exclusion chromatography (SEC) coupled to refraction index and multi-angle light scattering detectors (MALS), we determined the oligomeric state of full-length hHsp70 and delta-hHsp70. The three light scattering detectors exhibited a superposed signal characteristic of a monodisperse protein for both proteins. A single peak suggested homogenous samples in both cases (Fig. 4a, b). This last observation supports the notion that proteolysis occurs with a single cleavage resulting in delta-hHsp70. Moreover, the SEC-MALS spectra analysis showed a low percentage of aggregated forms (less than 1 %) for both proteins. This last observation supports the fact that the samples were properly folded and stable.

The recombinant full-length hHsp70 data analysis showed a single peak with a calculated mass from the MALS experiment of 139 kDa, corresponding to a hHsp70 dimer. The theoretical mass for a dimer is estimated at 140 kDa (Fig. 4a, b).

The molecular mass of delta-hHsp70 estimated by a MALS instrument was 59 kDa, thus suggesting a monomeric state of the protein. These data were in agreement with the results obtained by SDS-PAGE and mass spectroscopy fingerprinting, thereby suggesting the following: (i) a molecular mass of approximately 60 kDa and (ii) the homogeneity of the sample supports a single cleavage site leading to truncated hHsp70. The absence of major structural modifications between the full-length protein in the dimeric state and the truncated form in the monomeric state, as demonstrated by circular dichroism, suggest no major structural changes during the dimerization process. To further demonstrate the existence of a hHsp70:hHsp70 interaction, we have quantified the following protein-protein interactions: (1) hHsp70:hHsp70, (2) hHsp70:hHsp40, and (3) DnaK:DnaK with another technique: the Bio-Layer Interferometry (BLI). BLI is an optical- and label-free technique sensitive to an increase of mass bound to a biosensor (e.g., bound analyte). BLI provides sensorgrams like surface plasmon resonance. To perform the experiment, after biotynilation of hHsp70, the protein was immobilized onto the biosensor tip. Then, we tested the ability of hHsp70 to interact with hHsp70 in solution. These results clearly demonstrate that hHsp70 is interacting with hHsp70 in solution, demonstrating the capacity of hHsp70 to dimerize. The technique allows observing the small fraction of dimer exchanging their partner (supported by the 1:1 model fitting the sensograms). Moreover, hHsp70 presents a high-affinity in the low nanomolar range for itself in agreement with the ranges observed for dimers (*K*_*D*_ ~5 nM, Fig. 4c). We also show that, the well-known hHsp70:hHsp40 interaction has a greater affinity (*K*_*D*_ ~0.5 nM, Fig. 4d). Also, in line with the literature collated in Table 1, we confirm the DnaK:DnaK interaction (Fig. 4e), as a positive control of this experiment. Its affinity compares to the hHsp70:hHsp70 interaction (*K*_*D*_ ~5 nM).

**Table 1:** Studies detailing the oligomerization status of different Hsp70 family members along with the method used to determine the status

Hsp70 family	Quaternary structure/Oligomerization form (method)	Domain involved
DnaK (*E coli)*	Monomer (SEC-LS) (Kityk et al. 2012)	n.a.
	Monomer, mainly dimer, possibly trimer (SAXS, SEC) (Shi et al. 1996)	n.a.
	Forms monomers, dimers and higher molecular mass oligomers (DLS, SEC, native and SDS-PAGE) (Schonfeld et al. 1995)	
	An ensemble of monomers, dimers, and other small defined multimers (cross-linking, EM, native, and SDS-PAGE) (Thompson et al. 2012)	
BiP (*M. musculus)*	Monomer, dimer, and trimer (native PAGE) (Blond-Elguindi et al. 1993)	n.a.
Hsc70 *(rat)*	Monomer to at least trimer (cross-linking, native PAGE, SEC) (Benaroudj et al. 1995)	n.a.
	Monomer, dimer, and/or trimer (SEC) (Benaroudj et al. 1996)	n.a.
	Monomer and oligomer (SDS-PAGE, SEC) (Benaroudj et al. 1997) Oligomer, probably tetramer (SEC) (Fouchaq et al. 1999)	C-terminal
Hsc70 (*H. sapiens)*	Monomer, dimer, and trimer (cross-linking) (Kim et al. 1992)	n.a.
Hsp70 (*H. sapiens)*	HSP70 and HSC70 can form monomers, dimers, and oligomers (SDS-PAGE) (Angelidis et al. 1999)	n.a.
	Monomeric, dimeric, trimeric, and presumably oligomeric species (SEC, native and SDS-PAGE) (Nemoto et al. 2006)	C-terminal
	Forms monomers, dimers, trimers, and tetramers (DLS, SEC) (Aprile et al. 2013)	Linker + C-terminal SBD

*DLS* dynamic light scattering, *EM* electron microscopy, *LS* light scattering, *PAGE* polyacrylamide gel electrophoresis, *SAXS* small angle x-ray scattering, *SBD* substrate binding domain, *SDS* sodium dodecyl sulfate, *SEC* size exclusion chromatography, *n.a.* not available

**Fig. 5:**
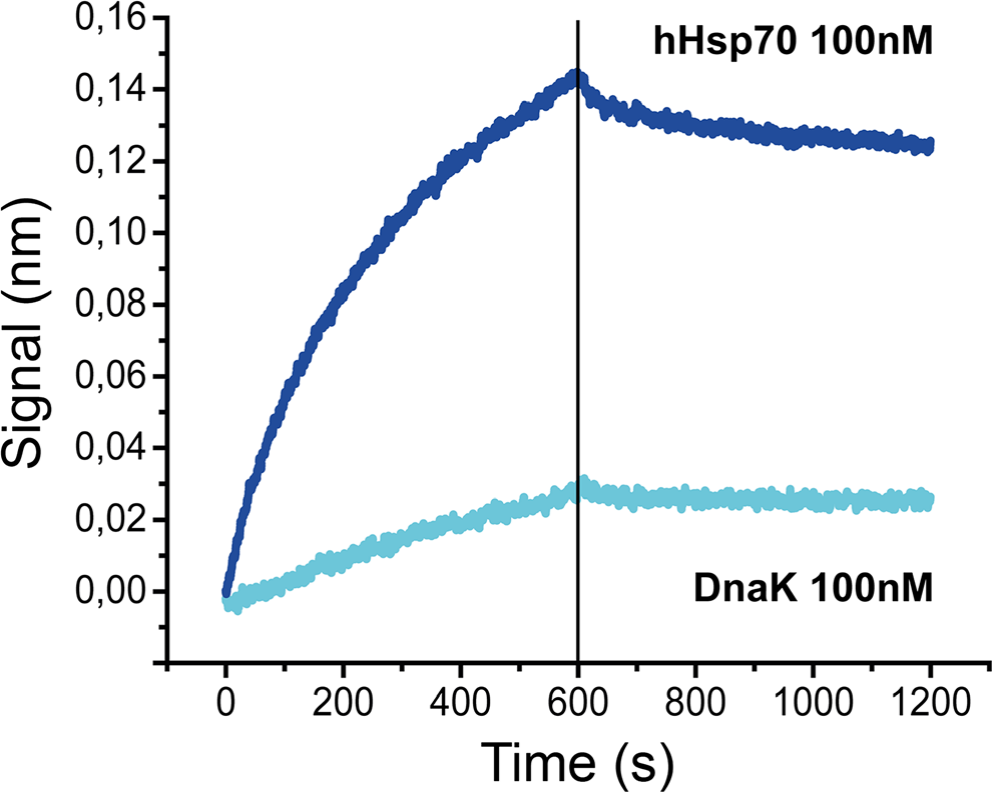
Sensorgrams representing the interaction between hHsp70 and the Hsp70 peptide aptamer A17. In contrast to the capture of hHsp70 at 100 nM with peptide aptamer A17 (*dark blue sensorgram*), the bottom sensorgram obtained by Bio-Layer Interferometry (*BLI*) (*light blue*) indicates that A17 and DnaK hardly interact. This stresses the actual differences in the N-terminal region of hHsp70 and DnaK, although this region is largely considered as well conserved. A17:DnaK binding fits with a 1:1 model (not excluding a 1:2 model) and A17:hHsp70 with 1:2 model (excluding the simplest 1:1 model). An estimation of the K_D_ is in the 50 nM range for A17:DnaK whereas an estimation of the K_D_ of A17:hHsp70 is around 6 nM

In another BLI experiment, we used a previously selected peptide aptamer (A17) arising from a screen for its ability to bind to the N-terminal domain of hHsp70 (Rerole et al. 2011). As shown in Fig. 5, the measured signal shows a binding of hHsp70 with the A17 peptide aptamer. This binding is only fit by a 1:2 (A17: hHsp70 × 2) binding model with a *K*_*D*_ in the low nM range. It corresponds to the affinity of the hHsp70 (as a homodimer) for the A17 peptide aptamer. This result supports that the protein is properly folded. Also, it indirectly shows, by this third method, the dimeric state of the protein. Interestingly, in contrast to the A17:hHsp70 interaction, the A17:DnaK interaction is quite weak (around 6 nM compared to 50 nM, respectively, Fig. 5). This further demonstrates the specificity of the A17 ligand for hHsp70.

### Functional assay

Two aspects of hHsp70 function, ATPase activity and refolding activity were monitored.

**Fig. 6:**
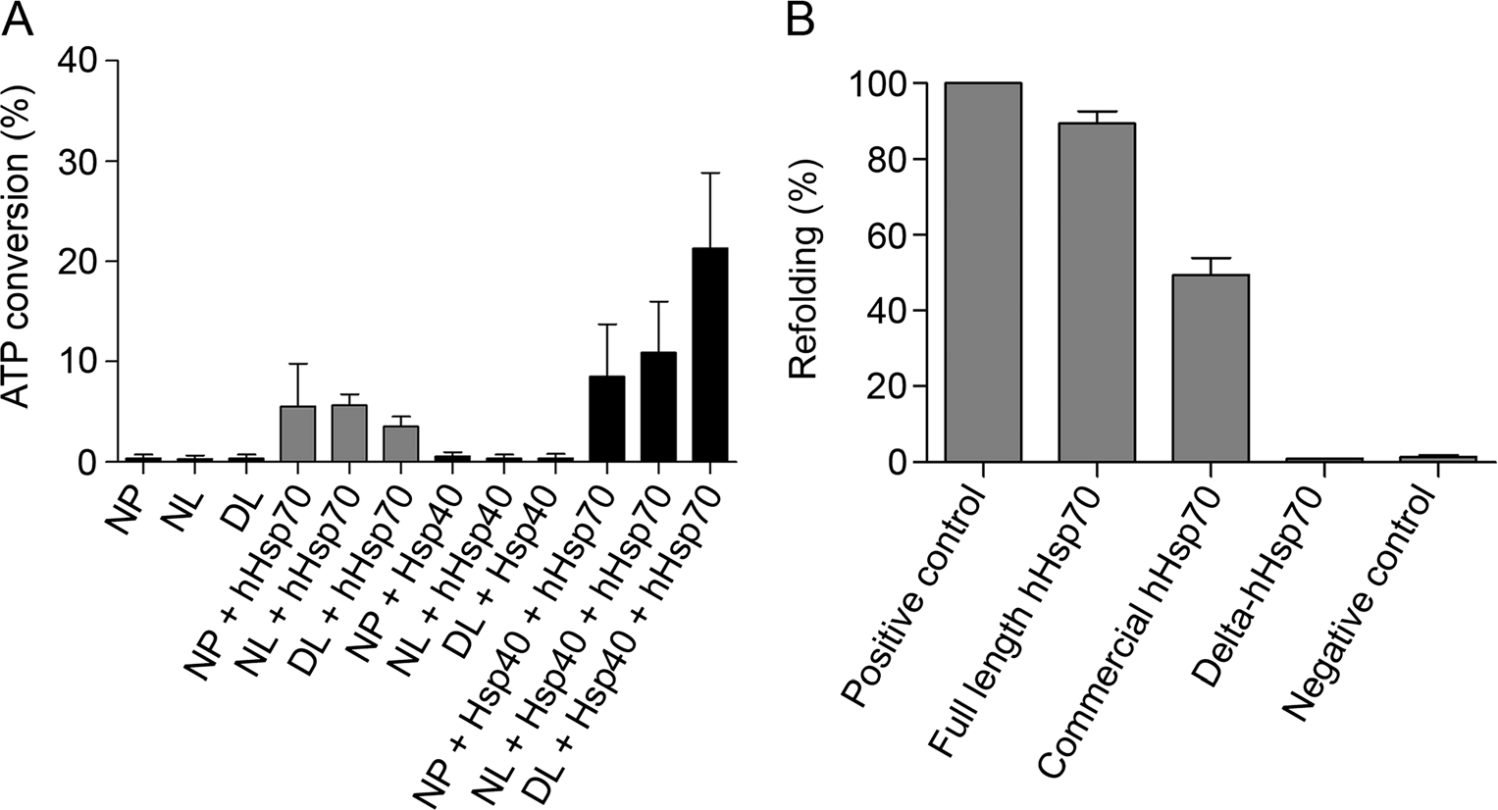
Confirmation of the chaperone function with a firefly luciferase refolding assay and ATPase activity assay. The *bar diagram* represents the percentage of ATP to ADP hydrolysis (**a**): with no proteins (*NP*), with native firefly luciferase (*NL*), or with denatured firefly luciferase (*DL*) in a refolding buffer containing either no Hsps, our full-length hHsp70 alone, hHsp40 alone, or the both Hsps. ADP formation was assessed by measuring the loss of d2-ADP fluorescence at 665 nm, reported by cryptate fluorescence at 620 nm after 1 h of incubation. The percentage of ATP to ADP hydrolysis values are obtained from an ATP/ADP standard curve. The *Pareto plot* represents the percentage of firefly luciferase refolding (**b**) in the presence of the full-length hHsp70, delta-hHsp70 (detailed in the present paper), and a commercially available hHsp70. The refolding of luciferase after denaturation (0.2 M guanidinium-HCl for 30 min at 25 °C) was measured by collecting total light at 560 nm after 3 h of incubation at 35 °C. Values are compared to a positive control (non-denatured luciferase only, set to 100 % of refolding) and a negative control (denatured luciferase incubated without Hsps)

ATPase activity was assessed by a homogeneous time-resolved fluorescence technique (HTRF) that follows the percentage of ATP to ADP enzymatic hydrolysis. The bar diagram represents the percentage of converted ATP (Fig. 6a): with no proteins (NP), with native firefly luciferase (NL), or with denatured firefly luciferase (DL) in a refolding buffer containing either no Hsps, our full-length hHsp70 alone, or with hHsp40. This data demonstrates the ATPase activity of hHsp70 compared to hHsp40. Moreover, this activity was dramatically increased in combination with its natural co-chaperone, hHsp40. Adding a native client protein increased this ATPase activity that was further increased when the client protein is denatured. This result demonstrates the active state of the purified protein and its efficient coupling to human Hsp40.

The refolding activity of the recombinant full-length hHsp70 and delta-hHsp70 proteins was then measured by their ability to refold a denatured firefly luciferase protein. In this colorimetric assay, different types of Hsp70 are combined, one at a time, with a human Hsp40. The refolding percentage is compared to the positive (non-denatured, thus luminescent firefly luciferase) and negative (denatured, thus non-luminescent firefly luciferase, without Hsp70) controls. In order to monitor refolding following denaturation, different kinetics (60 min, 120 min, 180 min) were investigated (data not shown). The best condition was obtained after a 180 min of refolding kinetics and used for all conditions. The Pareto plot (bar diagram in descending order) in Fig. 6b indicates that the full-length and non-tagged hHsp70 produced here presented a refolding activity when combined with Hsp40. The produced hHsp70 presented a refolding efficiency of 95 %. Interestingly, this activity was twofold higher compared to the commercially available hHsp70 (tagged). The delta-hHsp70 did not present any refolding activity. The lack of activity for this properly folded truncated protein can be explained by (i) the loss of dimerization ability, or (ii) the loss of an essential part of the protein for its chaperone activity, or (iii) both. As expected, the negative control presented a very low activity. In these conditions, this assay demonstrates the refolding activity (95 %) of the produced hHsp70.

## Discussion

### Validation of hHSP70 structure

In this paper, we report that hHsp70 can be expressed in milligram quantities using *E. coli*. The decrease of the fluorescence signal and the red shift of the emission frequency when a chaotropic agent is mixed with hHsp70 in solution support the quality of the purification. These modifications of the fluorescence are typically associated with the denaturation of a protein that was initially well-folded. The quantity of purified protein allows for the characterization of the secondary structures by the deconvolution of the CD spectra; the percentage of alpha-helix was estimated for the entire protein to be approximately 50–60 % and the percentage of beta-sheet to be approximately 25–30 %. The percentage of alpha-helix and beta-sheet estimated from the crystal structure of the homolog *E. coli* DnaK are 36 and 27 %, respectively (PDB ID: 2KHO) (Bertelsen et al. 2009). The proportion of secondary structure elements in hHsp70 and in *E. coli* DnaK are thus in the same range. Our experimental data for hHsp70 supports the previous model prediction of the secondary structure of hHsp70 (Nicolaï et al. 2013). Sparse evidences of the oligomerization status of some Hsp70 family members are collated in Table 1. In this study, the SEC-MALS analysis revealed a monodisperse homodimeric hHsp70 with a very low percent of aggregated forms. The BLI experiments clearly confirm with another approach the dimeric state of the protein and provides us with a *K*_*D*_ in the low nanomolar range (*K*_*D*_ ~5 nM) for the interaction between the two hHsp70 proteins forming the dimer.

### Validation of hHsp70 activity

The activity of the purified protein was tested with a refolding assay using firefly luciferase. The observed activity was 2 to 3 times higher compared to the commercially available His-tagged hHsp70. This result is consistent with a previous observation of activity alteration when hHsp70 is tagged (Boice and Hightower 1997). The present results demonstrate that the hHsp70 produced here adopts its full native state and is fully functional as shown by its chaperone as well as its ATPase activity.

### C-terminal part involved in hHsp70 dimerization

The BLI data, measuring the binding of hHsp70 with the A17 ligand (Fig. 5), demonstrates a high-affinity as the A17: hHsp70 interaction is around 6 nM. Besides, the comparison of all available model fittings is consistent with the homodimeric nature of hHsp70. This third proof is indirect but complementary to the two first evidences provided by the SEC-MALS data and the BLI determination of hHsp70: hHsp70.

Interestingly, we used a previously selected peptide aptamer (A17) for its ability to specifically bind to the N-terminal domain of hHsp70, further supporting the role of the C-terminal part in the dimerization. Although the N-terminal domain is generally considered to be the most conserved (compared to the C-terminal domain), our data enlightens actual differences between human and bacterial Hsp70 N-terminal regions (Aprile et al. 2013; Flaherty et al. 1990; Hartl and Hayer-Hartl 2009; Schmid et al. 1994; Zhang and Walker 1998).

Our peptide mass fingerprinting of the purified truncated form combined with the SEC-MALS analysis demonstrates the role of the C-terminal part in the dimerization (I562 to D641). Interestingly, this protease sensitive region has been previously reported for bovine Hsc70 (Chappell et al. 1987). Our observations of another Hsp70 family member raise the question of the physiological relevance of the truncation we present here for hHsp70 (cleavage site databases such as PeptideCutter and MEROPS did not allow us to identify a highly sequence-specific protease). Interestingly, delta-hHsp70 does not present chaperone activity. This observation can be explained by the truncation of the C-terminal part containing the EEVD motif. The EEVD motif has been demonstrated to be necessary for the interaction with the folding partner Hsp40 (Freeman et al. 1995). The C-terminal part identified in the homodimerization present two cysteine residues. The full-length hHsp70 was observed at the same apparent mass by SDS-PAGE when loading with or without reductant dithiothreitol treatment (data not shown). Recently, a study suggested the role of the interaction between the interdomain linker and the SBD in the oligomerization process (Aprile et al. 2013). This result together with our SDS-PAGE observations is in contrast with a previous study showing a disulfide bridge formed by cysteine 574 in the hHsp70 dimer (Nemoto et al. 2006). The last 80 C-terminal residues have been demonstrated to be involved in dimerization. Interestingly, the sequence identity of these 80 C-terminal amino acids with DnaK is approximately 20 %, whereas the global identity is calculated at 50 %. This observation warns us to be careful in generalizing the results for DnaK as it presents an EEVKD sequence, whereas hHsp70 bears the EEVD motif.

## Conclusion

In conclusion, this study provides strong evidence for the homodimeric nature of hHsp70 and identifies the last 80 C-terminal residues as involved in the dimerization process. Our experiments revealed that the dimerization process is governed by non-covalent interactions. As shown in the present paper, it is possible to purify large quantities of full-length non-tagged properly folded and active hHsp70, thereby opening up the possibility of the biophysical/biochemical studies presented in this study or radiocrystallography.

Although the physiological role of the hHsp70 homodimer remains to be elucidated, its dimeric nature may be a way to regulate its function, and potentially sets a new paradigm for human Hsp70.

## Materials and methods

### Construction of the hHsp70 expression plasmid

A codon-optimized gene encoding for hHsp70 expression in *E. coli* was synthesized (Geneart, Carlsbad, California). Nde1 and Sac1 restriction sites were inserted into the 5’ and 3’ ends, respectively, of the open reading frame coding sequence for hHsp70. The digested sequences were ligated in a pET21a plasmid previously opened by the same restriction enzymes in the cloning cassette. The resulting expression vector (pET21-hHsp70) encodes a full-length hHsp70 translated in the following sequence:

MAKAAAIGIDLGTTYSCVGVFQHGKVEIIANDQGNRTTPSYVAFTDTERLIGDAAKNQVALNPQNTVFDAKRLIGRKFGDPVVQSDMKHWPFQVINDGDKPKVQVSYKGDTKAFYPEEISSMVLTKMKEIAEAYLGYPVTNAVITVPAYFNDSQRQATKDAGVIAGLNVLRIINEPTAAAIAYGLDRTGKGERNVLIFDLGGGTFDVSILTIDDGIFEVKATAGDTHLGGEDFDNRLVNHFVEEFKRKHKKDISQNKRAVRRLRTACERAKRTLSSSTQASLEIDSLFEGIDFYTSITRARFEELCSDLFRSTLEPVEKALRDAKLDKAQIHDLVLVGGSTRIPKVQKLLQDFFNGRDLNKSINPDEAVAYGAAVQAAILMGDKSENVQDLLLLDVAPLSLGLETAGGVMTALIKRNSTIPTKQTQIFTTYSDNQPGVLIQVYEGERAMTKDNNLLGRFELSGIPPAPRGVPQIEVTFDIDANGILNVTATDKSTGKANKITITNDKGRLSKEEIERMVQEAEKYKAEDEVQRERVSAKNALESYAFNMKSAVEDEGLKGKISEADKKKVLDKCQEVISWLDANTLAEKDEFEHKRKELEQVCNPIISGLYQGAGGPGPGGFGAQGPKGGSGSGPTIEEVD

The integrity of the construct was verified by DNA sequencing.

### Production of hHsp70

BL21 Star™ (DE3), cells were transformed with the pET21-hHsp70 plasmid. The selected clones obtained on agar Luria broth (LB) plate supplemented with ampicillin (100 μg/L) after cell transformation were used to inoculate a 100-mL LB culture (100 μg/L ampicillin). After 12 h, 20 mL of culture was transferred to 1 L of LB culture (100 μg/L ampicillin) and incubated at 37 °C. Cell growth was stopped by centrifugation at 4,000×*g*, 20 h after induction by 1 mM (final concentration) IPTG.

### Purification of hHsp70

After production and centrifugation, cells were resuspended in buffer A (50 mM Tris–HCl, pH 8.0) supplemented with 20 μM of dithiothreitol. Cells were then disrupted by sonication at 4 °C and centrifuged at 17,000×*g* for 45 min at 4 °C. The supernatant cell lysate was loaded onto an 80-mL DEAE-Sepharose column (GE Healthcare). After washing with two column volumes of buffer A, proteins were eluted by a KCl linear gradient (0 to 1 M KCl in buffer A). A 40-mL Q-Sepharose column and a Superdex 200 Hiload 26/60 (GE healthcare, Little Chalfont, Buckinghamshire, UK) coupled to an AKTA FPLC were used for the following purification steps. Identical buffer, washing, and elution protocols were used on the Q-Sepharose and the DEAE column. Separation on the Superdex 200 was performed with an isocratic flow of buffer A. The protein concentration before size exclusion chromatography and after the last purification step was conducted using an Amicon Ultra (Millipore) filter unit with a cutoff of 10 kDa. The final pure protein fractions were pooled and concentrated to approximately 100 μM and either directly frozen at 20 °C or tested for activity. Purity of the proteins was assessed using SDS-PAGE with Coomassie-blue staining. Quantification of the full-length hHsp70 was determined using UV spectroscopy with a molar extinction coefficient of 33,350 M^−1^ cm^−1^ at 280 nm, calculated from the amino acid composition of the protein on ProtParam (http://web.expasy.org/protparam/). The same method was used to determine a molar extinction coefficient of 26,360 M^−1^ cm^−1^ at 280 nm, based on the mass spectroscopy protein length estimation. The theoretical isoelectric pH of the two forms was calculated using the ProtParam tool (http://web.expasy.org/protparam/). The purified and concentrated proteins at 10 μM were frozen at 20 °C in a 50-mM Tris–HCl pH 8.0 buffer.

### Intrinsic fluorescence measurement

Intrinsic fluorescence was measured using a Cary Eclipse spectrofluorimeter (Varian Instruments) equipped with magnetic stirrers and a Peltier temperature control unit. All experiments were conducted at 25 °C. The emission spectra were recorded between 310 and 450 nm after excitation at 295 nm. Both excitation and emission bandwidths were 5 nm. The spectra were recorded in a 2-mL cuvette of 1 cm path-length with a 5-μM protein concentration in buffer A or buffer A with 2 and 4 M guanidine chloride.

### Far-UV circular dichroism

Far-UV circular dichroïsm (CD) spectra were recorded using a JASCO J-815 spectropolarimeter equipped with a Peltier temperature control set at 25 °C. Using a 0.01-cm path-length quartz cell (Hellma), the protein samples spectra (5 μM in 20 mM Tris–HCl pH 8.0) were recorded with a scan speed of 50 nm.min^−1^ between 188 and 260 nm. Spectra were averaged over 10 scans and corrected by subtraction of the spectra acquired for buffer (20 mM Tris–HCl, pH 8.0) alone. CD measurements were normalized to protein concentration and presented as molar ellipticity deg.cm^2^.dmol^−1^ using the Jasco Spectra Analysis software. The spectra were decomposed to α-helix, β-sheet, β-turn, and random coil elements using Yang’s reference set (Venyaminov et al. 1993).

### Peptide mass fingerprinting

Purified protein band sequences were identified by peptide mass fingerprinting using tryptic cleavage combined with MALDI-ToF analysis. After purification, the full-length or the truncated forms of hHsp70 were loaded on SDS-PAGE. The corresponding bands were cut and extracted from the gel. After reduction and alkylation, they were digested with 100 ng of pig trypsin (PROMEGA). The peptides were injected on a LC-MS orbitrap. The PAPPSO platform (INRA, Jouy-en-Josas) was employed to conduct the protein identification using MS-Fit (Protein Prospector, UCSF).

### Size exclusion chromatography with on-line multi-angle light scattering (SEC-MALS)

The investigation of protein oligomeric states was performed on a Jasco PU-2080 Plus system consisting of a pump, a vacuum degasser, an autosampler, and a silica column MP015S5 or MP030S5 (7.8 mm, 300 mm), (Wyatt, Santa Barbara, CA). The MP015S5 column was used for the hHsp70 monomer, whereas the MP030S5 column was used for the hHsp70 dimer. The detection was carried out using a refractometer (RID-10A, Shimadzu) and a miniDAWN TREOS light scattering detector (Wyatt Technology). The column temperature was regulated at 20 °C. The buffer used for the isocratic gradient at 0.5 mL/min was a 0.1-M sodium phosphate buffer at pH 7.0. The 90° detector of the miniDAWN TREOS was calibrated using a toluene solution, the other detectors (45° and 135°) were normalized using a 10-mg/mL BSA solution in 0.1 M phosphate buffer, pH 7.0. The toluene calibration of the system was confirmed for different molecular sizes and oligomeric states of known proteins.

The solute concentration (C) was obtained directly from the refractive index signal for each volume slice according to C = (Vi K)/(dn/dc), where Vi is the detector output voltages for volume slice i, and K is the calibration constant. A dn/dc value of 0.186 mL/g was used for the calculations. The molecular mass of the protein was computed using the ASTRA V software (Wyatt Technology, Santa Barbara, CA, USA) based on the light scattering and refractive index signals.

### Bio-Layer Interferometry

Bio-layer interferometry (BLI), an optical and label-free technique sensitive to an increase of mass bound to the biosensor (e.g., bound analyte), provides sensorgrams as surface plasmon resonance. The first BLI experiment (Fig. 4), measures the affinity of the interacting protein. In a first step, the proteins (5 μg of biotinylated hHsp70 or DnaK) were incubated in a PBS buffer with a 1:3 ratio molar ratio of biotin. The free biotin was removed using a desalting column (Pierce). Then, the biotinylated protein was immobilized onto streptavidin biosensor tips and dipped into wells containing the analyte at different concentrations in PBS: hHsp70 (200, 300, 400, and 500 nM); hHsp40 (20, 30, 40, and 50 nM); and DnaK (200, 300, 400, and 500 nM). The second BLI experiment (Fig. 5) allowed the measurement of the dimeric hHsp70 interaction with a specific ligand. A17 binds to the N-terminal region of hHsp70 (Rerole et al. 2011; Seigneuric et al. 2011a) and was biotinylated with a 1:3 ratio (biotin-PEG4-NHS from Pierce EZ kit, prepared following the manufacturer’s instructions). The free biotin was removed using a desalting column (Pierce), then, the biotinylated ligand (A17) was immobilized onto streptavidin biosensor tips and dipped into wells containing the analyte (hHsp70 or DnaK at 100 nM in PBS). Both experiments (total volume in each well: 200 μL; shake speed: 1,000 rpm, association phase: 600 s; dissociation phase: 600 s) were background corrected, smoothed with the Savitzky-Golay algorithm and analyzed using OctetRED instrument software (ForteBio Data Analysis version 7.1.). For the first experiment (Fig. 4), all sensograms were fit with a 1:1 model. For the second type of experiment (Fig. 5), all available models were tested; but only the 1:2 model (bivalent analyte) was matching for the A17:hHsp70 interaction (excluding a 1:1 model). However, for the A17:DnaK interaction, the 1:1 model was matching (not excluding a more complex 1:2 model).

### hHsp70 ATPase activity assay

Firefly luciferase was denatured with 6 M guanidium-HCl, and then incubated with ATP (100 μM) and/or Hsp40 (160 nM) and/or hHsp70 (800 nM) for an hour. ATP to ADP conversion was monitored using the HTRF transcreener ADP (Cisbio assays, Codolet, France), according to the manufacturer’s instruction. Briefly, after refolding, 10 μL of refolding mix was incubated for 1 h with 37.5 μL 1X HTRF transcreener ADP enzymatic buffer, 5 μL of Anti-ADP antibody coupled to the HTRF donor Eu^3+^ cryptate, and 5 μL of d2-coupled ADP acceptor. ATP hydrolysis to native ADP will compete with d2-coupled ADP, inducing a loss of fluorescence transfert from the anti-ADP cryptate conjugated antibody to the d2 acceptor. Native ADP formation was thus assessed by measuring the decrease in d2-coupled ADP fluorescence at 665 nm, reported to the increase in cryptate emission at 620 nm, using a Wallac 1420 VICTOR3 luminometer. Delta F were obtained by calculating 10,000 × the 665/625 ratio, and total ATP conversion was obtained by comparing with Delta F distribution of an ATP/ADP standard curve that mimics the reaction (with total adenosine remaining constant throughout the range).

### hHsp70 refolding assay

Firefly luciferase (10 μM, Sigma-Aldrich) was denatured with 0.2 M guanidinium-HCl for 30 min at 25 °C. Refolding of the denatured luciferase (80 nM) at 35 °C was monitored during 3 h in the presence of ATP (1 mM) and/or Hsp40 (160 nM) and/or hHsp70 (800 nM) (i.e., the full-length or truncated forms produced by us or from Stressgen). Finally, D-luciferin (0.25 mM) was added, and after 10 min, the percentage of refolded luciferase was measured by collecting total light at 560 nm by a Wallac 1420 VICTOR^3^ luminometer. The positive control (non-denatured luciferase) was set to 100 %. The negative control consists of denatured luciferase in the folding buffer without Hsps. The guanidinium concentration used for the denaturation and the time used for the refolding were optimized for refolding efficiency.

## Electronic supplementary material

Below is the link to the electronic supplementary material.

ESM 1 (DOCX 1,090 kb)

## Abbreviations

hHsp70: human heat shock protein 70
hHsp40: human heat shock protein 40
NBD: nucleotide binding domain
SBD: substrate binding domain
BLI: Bio-Layer Interferometry
SEC: size exclusion chromatography
SEC-MALS: size exclusion chromatography multi-angle light scattering
Hsc: human heat shock cognate protein
