# Supplementary material for: C-terminal amino acids are essential for human heat shock protein 70 dimerization
Source: Cell Stress Chaperones. 2014 Jul 17;20(1):61–72. doi: 10.1007/s12192-014-0526-3 (PMC4255253; doi:10.1007/s12192-014-0526-3)
Supplement: Supplementary file 1 — (DOCX 1,090 kb) [file 12192_2014_526_MOESM1_ESM.docx]

**C-terminal amino acids are essential for human heat shock protein 70 dimerization**

Guillaume Marcion^1,2,#^, Renaud Seigneuric ^1,2,#,*^, Evelyne Chavanne ^2,3^, Yves Artur ^2,3^, Loïc Briand ^3^, Tarik Hadi^1,2^, Jessica Gobbo^1,2^, Carmen Garrido ^1,2,4,¶^, Fabrice Neiers ^2,3,¶,*^.

^1^INSERM, UMR 866, Dijon, France;

^2^Université de Bourgogne, Esplanade Erasme, Dijon, France;

^3^Centre des Sciences du Goût et de l'Alimentation, INRA UMR 1324, CNRS UMR 6265, Université de Bourgogne, France;

^4^Anticancer Center Georges François Leclerc, Dijon, France;

^#,¶^ these authors contributed equally to the work.

^*^ corresponding authors

**Correspondence should be addressed to: Neiers Fabrice, CSGA 17 rue Sully, 21000 Dijon, France, Tel.: +33380693202, Fax: +33380693225, Email: fabrice.neiers@u-bourgogne.fr; or Renaud Seigneuric, INSERM U866, 7 blv Jeanne d’Arc, 21000 Dijon, Tel.: +33380393417, Fax.: +33380393434, Email: renaud.seigneuric@u-bourgogne.fr.**


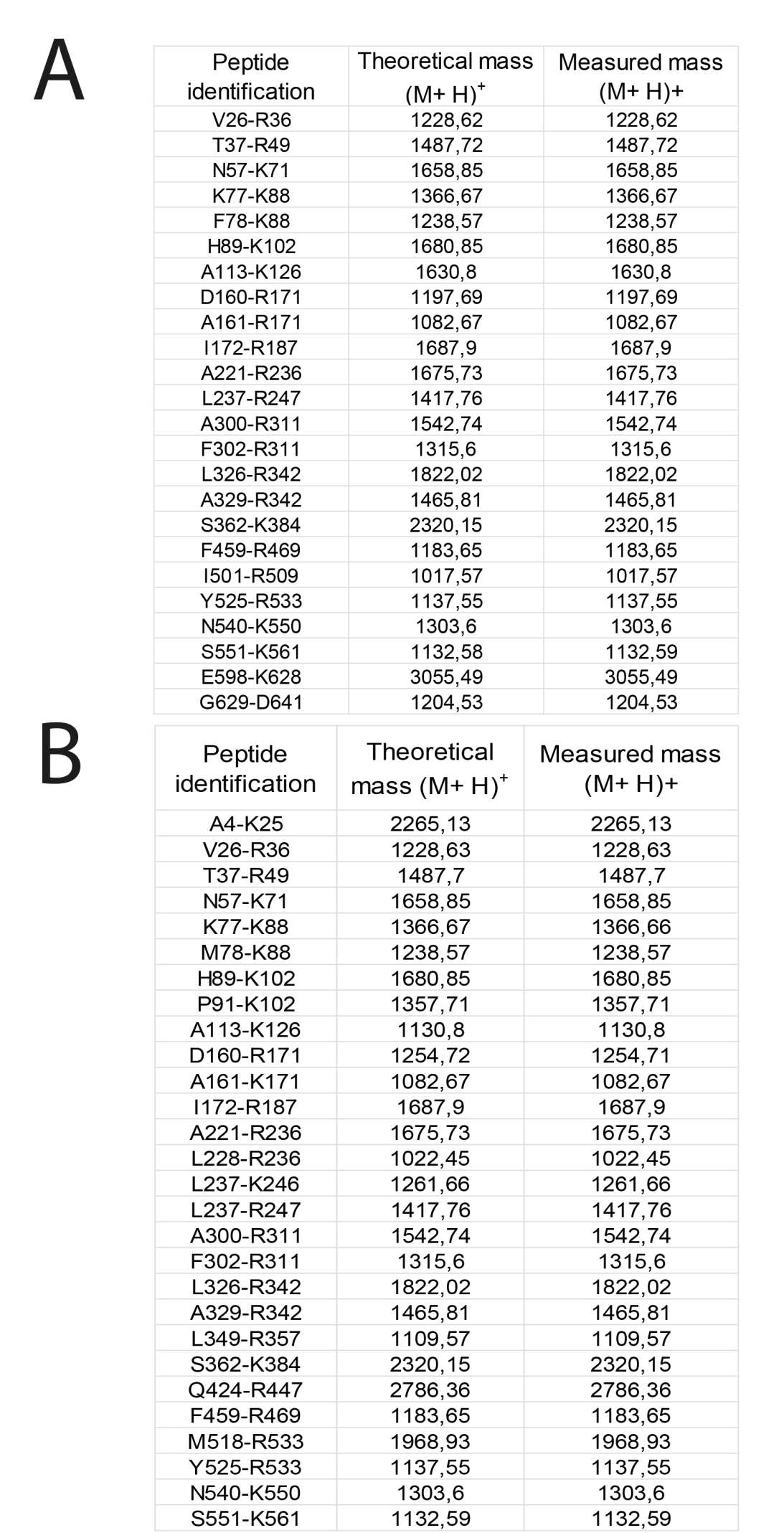


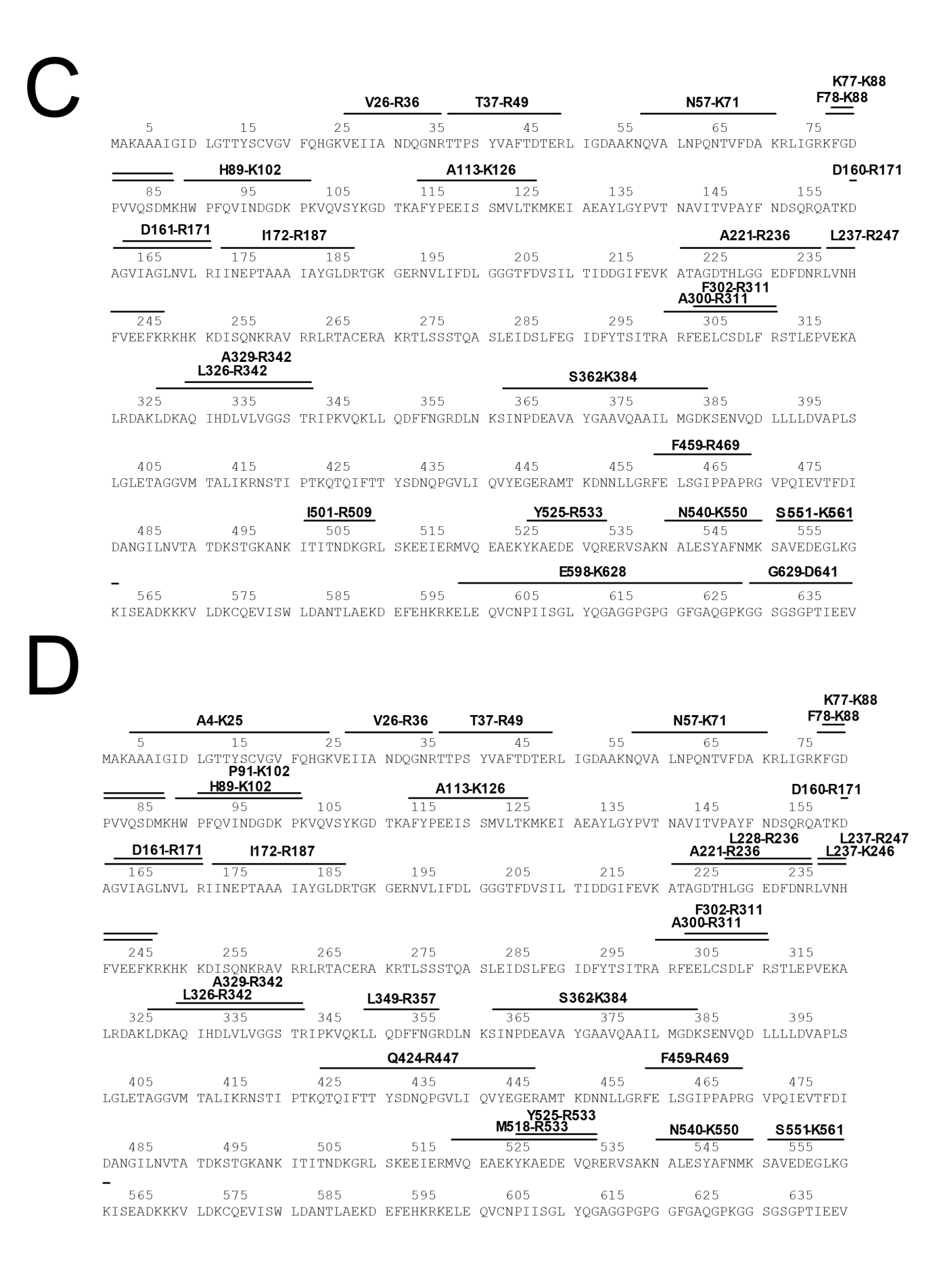


**Supplementary Fig. 1**

Purified hHsp70 band sequences identified by peptide mass fingerprinting using tryptic cleavage and coupled to MALDI-ToF analysis. Table S1 (A and B) represents the band sequences identified for the full-length hHsp70 and the delta-hHsp70, respectively. The results are shown on the sequence below for the full-length hHsp70 and the delta-hHsp70, respectively (C and D).
